# Effect of Endometriosis on Cumulus ATP, Number of Mitochondria and Oocyte Maturity in Cumulus Oocyte Complex in Mice

**DOI:** 10.1055/s-0043-1772186

**Published:** 2023-08-18

**Authors:** Muhammad Ardianta Widyanugraha, Widjiati Widjiati, Hendy Hendarto

**Affiliations:** 1 Department of Medical Science, Faculty of Medicine, Universitas Airlangga, Surabaya, Indonesia; 2 Department of Veterinary Science, Faculty of Veterinary Medicine, Universitas Airlangga, Surabaya, Indonesia; 3 Department of Obstetrics and Gynecology, Faculty of Medicine, Universitas Airlangga, Surabaya, Indonesia

**Keywords:** endometriosis, ATP, cumulus cells, mitochondria, oocyte, reproductive health

## Abstract

**Objective** Endometriosis causes a decrease in oocyte quality. However, this mechanism is not fully understood. The present study aimed to analyze the effect of endometriosis on cumulus cell adenosine triphosphate ATP level, the number of mitochondria, and the oocyte maturity level.

**Methods** A true experimental study with a post-test only control group design on experimental animals. Thirty-two mice were divided into control and endometriosis groups. Cumulus oocyte complex (COC) was obtained from all groups. Adenosine triphosphate level on cumulus cells was examined using the Elisa technique, the number of mitochondria was evaluated with a confocal laser scanning microscope and the oocyte maturity level was evaluated with an inverted microscope.

**Results** The ATP level of cumulus cells and the number of mitochondria in the endometriosis group increased significantly ( *p* < 0.05; *p* < 0.05) while the oocyte maturity level was significantly lower ( *p* < 0.05). There was a significant relationship between ATP level of cumulus cells and the number of mitochondrial oocyte ( *p* < 0.01). There was no significant relationship between cumulus cell ATP level and the number of mitochondrial oocytes with oocyte maturity level ( *p* > 0.01; *p* > 0.01). The ROC curve showed that the number of mitochondrial oocytes (AUC = 0.672) tended to be more accurate than cumulus cell ATP level (AUC = 0.656) in determining the oocyte maturity level.

**Conclusion** In endometriosis model mice, the ATP level of cumulus cells and the number of mitochondrial oocytes increased while the oocyte maturity level decreased. There was a correlation between the increase in ATP level of cumulus cells and an increase in the number of mitochondrial oocytes.

## Introduction

Endometriosis is defined as the presence of endometrial-like tissue outside the uterine cavity, which induces a chronic inflammatory reaction. 1 Approximately 30 to 50% of women with endometriosis have infertility and 25 to 50% of infertile women have endometriosis. 2

Although numerous potential explanations have been documented, the precise mechanism by which endometriosis results in infertility is not entirely understood. According to studies, endometriosis-affected women who undergo in vitro fertilization (IVF) had lower rates of fertilization and oocyte division than endometriosis-free women. 3 Oocyte quality in endometriosis also decreases. It has been demonstrated that GDF-9, a growth factor released by oocytes, is decreased in the follicular fluid of infertile endometriosis patients. 4 This is caused by inflammatory factors in the female pelvic area, where there is an increase in the concentration of inflammatory cytokines such as TNF-α which leads to impaired function or quality of oocytes indirectly through cumulus cells. 3 This is confirmed by a study by Nakahara et al., 5 which discovered that cumulus cells of endometrioma patients experienced a much higher rate of apoptosis than in patients without endometrioma.

Apoptosis in cumulus cells is believed to interfere with its function to support oocyte growth, which can eventually result in impaired oocyte maturation. As a result, essential nutrients like adenosine triphosphate (ATP) are primarily produced by cumulus cells to be supplied to oocytes 6 during oocyte maturation.

The nucleus and cytoplasm of the oocyte mature during the maturation process. The process of nuclear maturation involves the resumption of meiosis I, the ejection of the first polar body (Polar Body I), and the progression to the metaphase II (MII) stage. 7 Organelles like mitochondria are more numerous and are distributed differently in the cytoplasm. 8 Mitochondrial oocyte is the mitochondria present in the germ cell, oocyte, characterizing in oocytes by its rounder appearance and fragmented network. 9 Because mitochondria are not replicated during the cleavage stage of the embryo, the presence and quantity of mitochondria in mature oocytes are crucial for ensuring the survival of each blastomere that will obtain its mitochondria from the oocyte. 10 In both human and experimental animals, abnormal mitochondrial quantity is associated with poor oocyte quality. 11 12 13

One of the most crucial organelles in determining the quality of an oocyte is the mitochondria, which control the potential of oocyte growth through a variety of routes including ATP synthesis, Ca ^2+^ regulation, and maintenance of intracellular redox potential (IRP). The fact that mitochondrial oocytes grow in quantity, display extraordinary mobility, and cluster in a specific area, which is assumed to fulfill high energy demands during oocyte maturation is evidence for the crucial function that mitochondria play in oocyte and embryo development. 9 To divide, mitochondria require ATP compounds, which are taken from the cytoplasm. Oocytes use a large amount of their energy source (ATP) derived from cumulus cells for their maturation process and also increase the number of mitochondria during the maturation process because mitochondrial oocytes are in a “quiet” metabolic state or low activity to reduce their oxidant production. 14 The quantity of mitochondrial oocytes is believed to be affected by the decline in ATP supply from cumulus cells.

The aim of the present study was to analyze the effect of endometriosis on cumulus cell ATP level, the number of mitochondria and the oocyte maturity level. The present study used oocytes in mice as endometriosis model since studying the effect of endometriosis on oocytes in humans has ethical constraints.

## Methods

The present study had received ethical clearance from the Animal Care and Use Committee Faculty of Veterinary Medicine Universitas Airlangga, Surabaya, Indonesia (number 2.KE.134.12.2021). All procedures done in the present study were conducted in accordance to the United Kingdom Animal Act 1986. All surgeries were conducted under anesthesia and all efforts were made to minimize the suffering.

The present study had a true experimental design conducted in the laboratory on mice ( *Mus musculus* ). All experimental animals were randomly selected so that they had the same opportunity to receive treatment and had a control group. The sample size was determined using the Lemeshow formula (with a significance level of α = 0.05). Considering the average count of the control and treatment groups and the possibility of dropping out, the sample size was 16 mice per group.

The mice were obtained from a mouse breeder center for research purposes from the Integrated Research and Testing Laboratory (Laboratorium Penelitian dan Pengujian Terpadu, LPPT), Gadjah Mada University, Yogyakarta, Indonesia. Mice used in the study were healthy mice aged ± 12 weeks old, never pregnant, with a weight ranging from 20 to 30 g. Healthy mice are mice that based on clinical examination have bright eyes, no dirt, the fur is clean, not dull and not easy to fall off, no wound, and not aggressive.

In the present study, the experimental animal studies were prepared first. The mice were acclimatized for 1 week in a clean cage, with enough air, light, and homogeneous food and drink. Then, the mice were randomly divided into 2 groups, the control group (P0) and the endometriosis group (P1). To determine the sample size, the Lemeshow formula was used, with the result that each group consisted of 16 mice. 15

The making of endometriosis model mice was in accordance with what was done in the study by Hendarto. 16 In the present study, the presence of endometriosis was confirmed by the formation of endometriotic lesions and visually visible hypervascularization of the peritoneal tissue at surgery. Another visible sign was the attachment of the uterus and digestive tract or other organs. The mice were given an injection of cyclosporin A with the aim of suppressing their immune status, which was done intramuscularly on the thighs of the mice. The drug preparation available in Indonesia was Sandiimun produced by Novartis in 1 ampoule containing 50 mg/mL. The required dose was 10 mg/kg. In the present study, the weight of the mice ranged from 20 to 30 g, so the dose of cyclosporin A given must be adjusted. After calculating the dosage of the preparation, each mouse received a conversion dose of 1.8 mg/mouse so that the dose after dilution with water for injection was 0.2 mL of Sandiimun for each mouse. Endometriosis is induced by injecting endometrial tissue intraperitoneally and then by injecting estrogen to stimulate its growth. Endometrial tissue was taken from benign tumor uterine material, which was stored in phosphate buffered saline (PBS). Washing was performed 2 × with a centrifugal device with a rotation of 2,500 rpm. The supernatant was discarded and then PBS, penicillin 200 IU/ml and streptomycin 200 µg/ml were added. Then the wet endometrial tissue was taken with a 3 ml syringe. The dose given to mice was 0.1 ml. In addition, 54 IU estrogen injections (equivalent to 5.4 ug) were also given on days 1 and 5. In the control group, the mice received distilled water injections.

After 2 weeks, all groups of mice were given an injection of 5 IU Pregnant Mare Serum Gonadotropin (PMSG) hormone and 48 hours later they were injected with Human Chorionic Gonadotropin (hCG) and mixed with vasectomized male mice for ovulation induction. Seventeen hours after the mice mated, vaginal plug examination was performed. Female mice with positive vaginal plugs were terminated. Then, preparations for removing the fallopian tube organs were performed. The cumulus oocyte complex (COC) was collected by tearing the fertilization sac of the fallopian tube, which was done by observing the fallopian tube under a microscope. The COC was collected and the separation was performed using 0.1% hyaluronidase.

Adenosine triphosphate level on the cumulus cells obtained was then examined using the Elisa technique according to the instructions of the manufacturer. Measurement of ATP level was performed using Mouse Adenosine Triphosphate ELISA Kit, (Cat. No. E0665Mo, Bioassay Technology Laboratory) and it was performed with absorbance readings at a wavelength of 450 nm. The oocytes obtained were then examined for the number of mitochondria using the mito-tracker green (MTG) FM 50 μg from Invitrogen (then measured with a confocal laser scanning microscope (CLSM). The data obtained were then processed using special software Olympus Fluoview Ver.4.2a in a computer. The oocyte maturity level was evaluated using an inverted microscope with 200x magnification. The sign of oocyte maturity was determined when it reached the Metaphase II stage with polar body I removed.

**Fig. 1:**
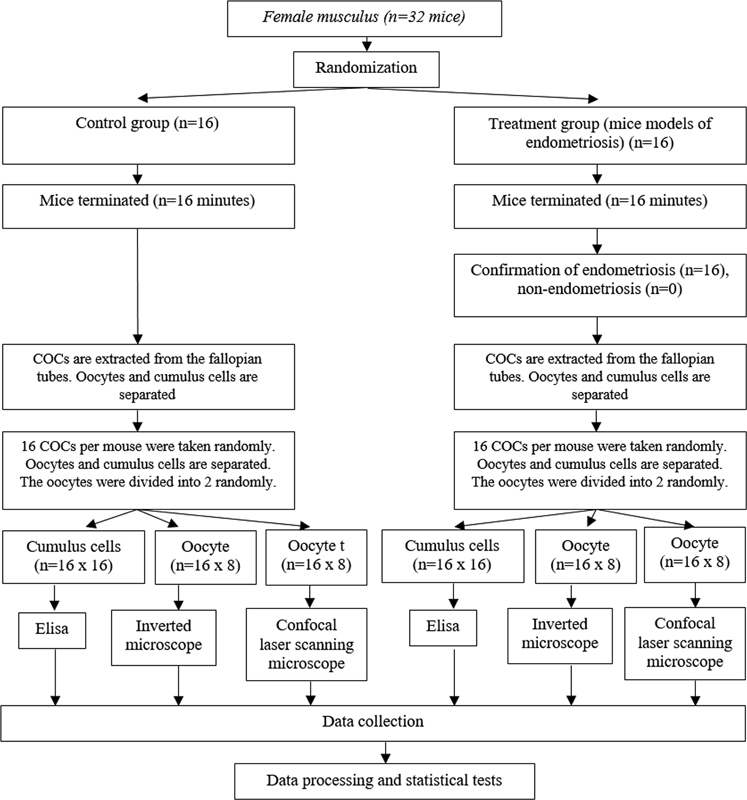
Research flow.

All statistical analyzes were performed using IBM SPSS Statistics for Windows version 26 (IBM Corp., Armonk, NY, USA). Data distribution was assessed by the Kolmogorov-Smirnov normality test. To determine the difference between the endometriosis group and the control group, a continuous dataset that was not normally distributed used the two-tailed Mann-Whitney U test and data that were normally distributed used the *t* -test. Correlation analysis of each variable used the Spearman or Pearson test, depending on the distribution of the data. A p-value < 0.05 was considered statistically significant. Receiver operating characteristic (ROC) curve analysis was performed to determine the sensitivity, specificity, and limit value of cumulus cell ATP levels or the number of oocyte mitochondria to oocyte maturation level ( Fig. 1 ).

## Results

**Fig. 2:**
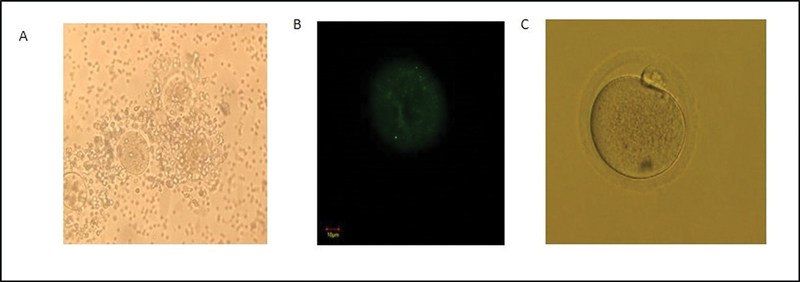
(A) Mice cumulus-oocyte complex (microscope magnification 40x). (B) Mitochondria on examination with CLSM (marked in green) (C) Mature oocytes on examination with an inverted microscope (200x magnification).

All 16 mice from the P0 group and 16 mice from the P1 group were found to have ovulation. All COC were collected, and oocytes were separated from cumulus cells that surrounded them ( Table 1 ) ( Fig. 2 ).

**Table 1:** Differences between the endometriosis group and the control group

	Group	Mean	SD or mean rank	*p* - *value*
ATP Level of mice cumulus cell in each group	P0 P1	25.84 30.15	1.97* 1.76*	0.000
The number of mitochondria in each group.	P0 P1	208,875 406,503	10.19** 22.81**	0.000
Oocyte maturity level	P0 P1	36.27 19.91	0.18* 0.16*	0.006

Abbreviations: ATP, adenosine triphosphate; SD, standard deviation.

** Mean rank.

The ATP level of all mice cumulus cells obtained was measured. The results of the *t* -test on ATP level of the two groups showed a significant difference ( *p* < 0.05) with ATP level of the control group significantly lower than that of the treatment group (endometriosis) with the mean in the control group of 25.84 ng/ml and in the endometriosis group of 30.15 ng/ml ( Table 1 ). We counted the number of mitochondria from oocytes taken from cumulus cells using CSLM. The Mann-Whitney test results on the number of mitochondria in the two groups revealed a significant difference ( *p* < 0.05), with the number of mitochondria in the control group significantly lower than that in the treatment group (endometriosis), with the means of the two groups being 208,875 and 406,503.25, respectively ( Table 1 ). We also assessed the oocyte maturity using an inverted microscope. According to the results of the *t* -test on the maturity level of the two groups, the oocyte maturity level in the control group was significantly higher ( *p* < 0.05) than that in the treatment group (endometriosis), with the mean in the control group being 36.27% and that in the endometriosis group being 19.91% ( Table 1 ). Then, using correlation test, we examined the relationship between these variables. We first examined the relationship between the ATP level in cumulus cells and the number of mitochondrial oocytes. The Spearman correlation test results revealed a significant relationship ( *p* < 0.01) between the ATP level in cumulus cells and the number of mitochondrial oocytes, in which the ATP level in cumulus cells had 46% influence on the number of mitochondrial oocytes ( Table 2 ).

**Table 2:** The effect of ATP level in cumulus cell on the number of mitochondrial oocytes, the effect of the number of mitochondrial oocytes on oocyte maturity level, and the effect of cumulus cell ATP level on the oocyte maturity level

	r	*p* - *value*
Effect of cumulus cell ATP levels on the number of oocyte mitochondria	46%	0.008
Effect of the number of oocyte mitochondria on the level of oocyte maturity.	−	0.102
Effect of cumulus cell ATP levels on oocyte maturity level.	−	0.381

Abbreviations: ATP, adenosine triphosphate.

Second, we examined the correlation between the number of mitochondrial oocytes and the level of oocyte maturity. According to the results of the Spearman correlation test ( *p* > 0.01), there was no significant correlation between the number of mitochondrial oocytes and the level of oocyte maturity ( Table 2 ). The correlation between the ATP level in cumulus cells and the oocyte maturity was then examined. The findings of the Pearson correlation test revealed that there was no significant correlation between the number of mitochondrial oocytes and ATP levels in the cumulus cells (p > 0.01) ( Table 2 ). Finally, we also performed further analyses to assess the diagnostic value of cumulus cell ATP level or mitochondrial number or oocyte maturity level. The results of the ROC curve revealed no significant correlation between ATP level in cumulus cells or the number of mitochondrial oocytes on the level of oocyte maturity. However, the number of mitochondrial oocytes (AUC = 0.672) was more accurate in predicting the oocyte maturity level than the ATP level of cumulus cells (AUC = 0.656) ( Fig. 2 ).

**Fig. 3:**
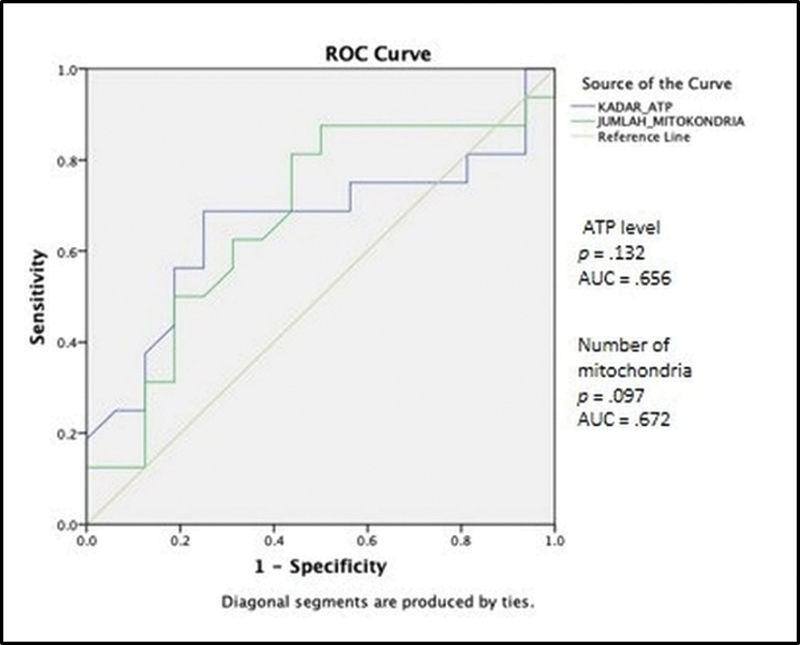
ROC curve of cumulus cell ATP level and the number of mitochondrial oocytes on the oocyte maturity level.

Fig. 3 shows the ROC curve of cumulus cell ATP level and the number of mitochondrial oocytes on the oocyte maturity level. The sensitivity and specificity of the cumulus cell ATP level and the number of mitochondrial oocytes were plotted using ROC analysis between the endometriosis group and the control group. The area under the curve (AUC) and the p-value are shown. The cumulus cell ATP level or the number of mitochondria were not proven to significantly differentiate the oocyte maturation level of the endometriosis group and that of the control group ( *p* = 0.132 and 0.097).

## Discussion

The results showed that ATP level in the endometriosis group significantly increased compared with that in the control group ( *p* < 0.05). This result is different from the study by Hsu et al., where the ATP levels of cumulus cells were lower in the endometriosis group. 17 This might be because this study was conducted in animal models where exposure to endometriosis was given in a shorter time, in contrast with women with endometriosis, who may take longer without knowing for sure when the exposure to endometriosis started. Another possibility is that the apoptosis that occurred in cumulus cells was incomplete or still at an early stage when the viability of cumulus cells was still > 50%. 18

Apoptosis is a form of cell death that requires energy. This is supported by a study by Zamareva et al. 18 Apoptotic stimuli, like TNF-α, cause cytoplasmic ATP levels to rise significantly. Excess cytoplasmic ATP triggers apoptotic execution events including caspase activation and DNA fragmentation. Therefore, an elevated level of cytoplasmic ATP is a prerequisite for apoptotic cell death. According to a study by Zamaraeva et al., 18 while cell viability remained at 50%, ATP signaling in the population of cells that underwent apoptosis returned to levels similar to those of the control group. Subsequently, cytoplasmic ATP signaling gradually decreased, which was accompanied by an increase in the number of dead cells. 15 The fact that cumulus cells naturally proliferate throughout the process of folliculogenesis under the influence of FSH, estradiol, 19 and growth factors generated by oocytes supports this explanation even more (GDF9, BMP15). 20 In a study by Hendarto et al., 4 it was discovered that patients with endometriosis had lower levels of GDF-9 in their follicular fluid. This suggests that cumulus cell proliferation may be declining, which confirms the finding of the study that the apoptotic process increased ATP levels.

The findings of our study also showed that there were significantly more mitochondrial oocytes in the endometriosis group than in the control group ( *p* < 0.05). This could be as a result of an increase in ATP supply from cumulus cells, which during the oogenesis process stimulates an increase in the number of mitochondria in the oocyte.

Oocytes in the germinal vesicle (GV) stage during follicular development interact closely with granulosa cells via transzonal projections (TZPs). It is well known that the oocyte lacks the enzymes for glycolysis, cholesterol biosynthesis, and receptors for specific amino acids, thus the metabolism of granulosa cells is modulated to meet the needs of the oocyte (through oocyte-derived factors, the GDF9, BMP15, and FGF8B). Adenosine triphosphate, pyruvate, amino acids, and cholesterol must therefore be captured or produced by granulosa cells and supplied to oocytes. 6 21 An essential molecule for mitochondrial division, DYNAMO1, known as the mitochondrial division machine (MD), is also involved in the process of increasing the number of mitochondrial oocytes at this stage. During mitochondrial division, DYNAMO1 uses cytoplasmic ATP as an energy source for membrane cleavage. 22 Therefore, the oocyte receives a larger energy supply along with an increase in ATP level in cumulus cells. This could activate the mitochondrial cleavage machinery to promote mitochondrial replication inside the oocyte.

Furthermore, cells with a high demand for energy, like neuron and muscle cells, tend to have greater numbers of mitochondria. 23 Since mature oocytes show a remarkable increase in the number of mitochondria, this suggests that the oocyte maturation process requires a large amount of energy. The consequence is that higher free radicals will be formed as a side effect of oxidative phosphorylation, which can trigger oxidative stress and be harmful to these cells. 24 However, it seems that the oocyte develops a protective mechanism by using most of its energy source (ATP) derived from cumulus cells for its maturation process so that the mitochondria of the oocyte are in a “quiet” metabolic state or low activity to reduce their oxidant production. 1 However, it is known that oxidative stress occurs in endometriosis, which is considered to be the cause of a decrease in oocyte quality, but the mechanism is not widely understood. 25 As a consequence, oocytes are thought to require greater energy needed to repair themselves than for the maturation process. However, further studies are needed to confirm this.

The findings of the present study were different from those of Sanchez et al., who claimed that the number of mitochondrial oocytes in the endometriosis group was lower than that in the group with infertility caused by factors other than the endometrium. 26 It should be emphasized that a decrease in the amount of mtDNA molecules – which are a component of the mitochondria and can contain several mtDNA molecules – confirmed the findings of this paper. In contrast to this study, the measurements were made based on the binding of MTG with certain compounds in the mitochondrial matrix that supported the alkylation of thiol groups available in this subcellular compartment.

Another possible explanation for the aforementioned differences was the presence of polymorphisms in mitochondria. Polymorphisms often occur in mitochondria and mitochondrial polymorphisms influence the pathophysiology of various diseases by influencing mitochondrial matrix pH and intracellular calcium dynamics. 26 27 Free thiols are six times more reactive in the mitochondria than in the cytosol because the pH in the mitochondrial matrix is higher than in the cytosol (7.8–8 versus 7.2). 28 Changed pH in the matrix has the potential to affect the results of the study. However, further studies are required to confirm this polymorphism.

We used a correlation test to see whether there was a relationship between an increase in the ATP level of cumulus cells and an increase in the number of mitochondrial oocytes. The results revealed a significant correlation ( *p* < 0.01) between the two variables. As a result, it is possible to infer that the ATP level in cumulus cells affects how many mitochondrial oocytes are present. The number of mitochondrial oocytes increases along with the ATP concentration of the cumulus cells. The higher the ATP content of the cumulus cells, the higher the number of mitochondrial oocytes. This supports the notion that mitochondria utilize the ATP present in the cytoplasm around them as a source of energy for reproduction. 22

The oocyte maturity level in the endometriosis group was significantly lower than that in the control group ( *p* < 0.05). This was highly contradictory when compared with the results of the aforementioned study that found an increase in the number of mitochondrial oocytes. With the abundance of mitochondria in the endometrial oocyte, the oocyte should have sufficient and independent resources to continue its maturation once its relationship with cumulus cells is damaged following the luteinizing hormone (LH) surge. This may be because there was a quantitative, instead of qualitative, increase in the number of mitochondria. This was supported by the correlation test we conducted between the number of mitochondrial oocytes and the maturity level of oocytes with the result that there was no significant relationship between the number of mitochondrial oocytes and the oocyte maturity level ( *p* > 0.01).

A possible explanation related to the aforementioned results is the emergence of free radicals as a side effect of ATP formation in cumulus cells. Free radicals (ROS) are a side effect of oxidative phosphorylation in the mitochondrial cells that are actively producing ATP. 29 Endometriosis can trigger destructive apoptosis and increased ROS through mitochondrial dysfunction of cumulus cells. 30 Mitochondrial ROS can damage DNA by producing various DNA damages such as oxidized bases and DNA chain severance. 30 31 Thus, in addition to ATP, ROS formed in cumulus cells also increased and consequently was distributed into oocytes through TZPs and caused damage to mtDNA. Therefore, when the mitochondrial oocyte is active, it cannot function properly to support the final maturation process of meiosis II and also the early development of the embryo, which requires energy. From this, oocyte mitochondrial dysfunction that initially does not appear to predispose to early oogenesis (before the LH surge) may eventually impair oocyte maturation, leading to infertility. 32 33 34 However, further studies are required to confirm this.

Another possible explanation is that there was an increase in free radicals in endometriosis due to iron overload that exceeds the storage capacity of macrophages. 35 According to Carlberg et al., 3 the peritoneal fluid of women with endometriosis contains inflammatory substances that can diffuse into the ovarian follicles. These free radicals will diffuse into the follicles, which contain the oocytes, exposing the mtDNA and causing damage.

An interesting concept was presented by Chiaratti et al., who stated that mitochondrial oocytes are in a “quiet” metabolic state or low activity. 9 Thus, why do oocytes store so many mitochondria if they are not required for oogenesis? The function of these organelles during the late maturation and early embryogenesis periods can, at least in part, provide an explanation for this. During early oogenesis, mitochondrial oocytes are relatively unnecessary because their cooperation with granulosa cells is sufficient to support the needs of the oocyte. As is widely known, the process of oxidative phosphorylation in mitochondria has a side consequence of free radical generation, and this incidence demonstrates the significance of protecting mitochondria from oxidative damage that results in mutations in mitochondrial DNA (mtDNA). 36 Given that mitochondria are inherited exclusively by the mother, oocytes with mtDNA mutations can cause disease in their offspring. Thus, to compensate for the expansion of mutations, oocytes have developed special mechanisms to protect the mtDNA molecule from damage. 2

Additionally, the correlation between cumulus cell ATP level and oocyte maturity level was also examined in the present study, and no significant correlation was found. This confirmed the theory that granulosa cells promote oocyte maturation prior to the LH surge. After the LH surge, the oocyte continues to mature on its own, using its own organelles. This was also confirmed in the present study, although there was no significant correlation between ATP level of cumulus cells or the number of mitochondrial oocytes with oocyte maturity level, the results of ROC curve for cumulus cell ATP levels or the number of mitochondrial oocytes on oocyte maturity level indicated that the number of mitochondrial oocytes (AUC = 0.672) tends to be more accurate than cumulus cell ATP level (AUC = 0.656) in determining the oocyte maturity level.

However, the limitation of the present study is the use a mouse model of endometriosis that was treated for 2 weeks. Endometriosis is a disease that induces a chronic inflammatory reaction. This research might be more suitable if the inflammatory conditions that occur are made longer.

## Conclusion

There was an increase in cumulus cell ATP level and in the number of mitochondrial oocytes in endometriosis model mice and a decrease in oocyte maturity level in endometriosis model mice. The increase in ATP level of cumulus cells corresponded to an increase in the number of mitochondrial oocytes. There was no significant relationship between cumulus cell ATP level and the number of mitochondria on the oocyte maturity level in metaphase II (PbI) although the number of mitochondrial oocytes tended to be more accurate in determining oocyte maturity level than ATP level of cumulus cells.

## References

[1] Chiaratti M R Garcia B M Carvalho K F Oocyte mitochondria: role on fertility and disease transmission. Proceedings of the 32nd Annual Meeting of the Brazilian Embryo Technology Society (SBTE) ; Florianopólis, SC, Brazil, August 16 ^th^ to 18th, 2018 10.21451/1984-3143-AR2018-0069 PMC8202466 34178146

[2] Missmer S A Hankinson S E Spiegelman D Barbieri R L Marshall L M Hunter D J Incidence of laparoscopically confirmed endometriosis by demographic, anthropometric, and lifestyle factors Am J Epidemiol 2004 160 08 784 796 10.1093/aje/kwh275 15466501

[3] Carlberg M Nejaty J Fröysa B Guan Y Söder O Bergqvist A Elevated expression of tumour necrosis factor alpha in cultured granulosa cells from women with endometriosis Hum Reprod 2000 15 06 1250 1255 10831550 10.1093/humrep/15.6.1250

[4] Hendarto H Prabowo P Moeloek F A Soetjipto S Growth differentiation factor 9 concentration in the follicular fluid of infertile women with endometriosis Fertil Steril 2010 94 02 758 760 19931079 10.1016/j.fertnstert.2009.10.011

[5] Nakahara K Saito H Saito T Ito M Ohta N Takahashi T Hiroi M Ovarian fecundity in patients with endometriosis can be estimated by the incidence of apoptotic bodies Fertil Steril 1998 69 05 931 935 10.1016/s0015-0282(98)00038-7 9591505

[6] Su Y Q Sugiura K Wigglesworth K O'Brien M J Affourtit J P Pangas S A Oocyte regulation of metabolic cooperativity between mouse cumulus cells and oocytes: BMP15 and GDF9 control cholesterol biosynthesis in cumulus cells Development 2008 135 01 111 121 10.1242/dev.009068 18045843

[7] Tripathi A Kumar K V Chaube S K Meiotic cell cycle arrest in mammalian oocytes J Cell Physiol 2010 223 03 592 600 10.1002/jcp.22108 20232297

[8] Collado-Fernandez E Picton H M Dumollard R Metabolism throughout follicle and oocyte development in mammals Int J Dev Biol 2012 56(10-12):799 808 10.1387/ijdb.120140ec 23417402

[9] Chiaratti M R Garcia B M Carvalho K F Macabelli C H Ribeiro F KS Zangirolamo A F Oocyte mitochondria: role on fertility and disease transmission Anim Reprod 2018 15 03 231 238 10.21451/1984-3143-AR2018-0069 34178146 PMC8202466

[10] Dumollard R Duchen M Carroll J The role of mitochondrial function in the oocyte and embryo Curr Top Dev Biol 2007 77 21 49 10.1016/S0070-2153(06)77002-8 17222699

[11] Duran H E Simsek-Duran F Oehninger S C Jones H W Jr Castora F J The association of reproductive senescence with mitochondrial quantity, function, and DNA integrity in human oocytes at different stages of maturation Fertil Steril 2011 96 02 384 388 10.1016/j.fertnstert.2011.05.056 21683351

[12] Lee S K Zhao M H Kwon J W Li Y H Lin Z L Jin Y X The association of mitochondrial potential and copy number with pig oocyte maturation and developmental potential J Reprod Dev 2014 60 02 128 135 10.1262/jrd.2013-098 24492657 PMC3999391

[13] Wai T Ao A Zhang X Cyr D Dufort D Shoubridge E A The role of mitochondrial DNA copy number in mammalian fertility Biol Reprod 2010 83 01 52 62 10.1095/biolreprod.109.080887 20130269 PMC2888963

[14] Chiaratti M R Garcia B M Carvalho K F Machado T S Ribeiro F KDS Macabelli C H The role of mitochondria in the female germline: Implications to fertility and inheritance of mitochondrial diseases Cell Biol Int 2018 42 06 711 724 10.1002/cbin.10947 29418047

[15] Dwiningsih S R Darmosoekarto S Hendarto H Dachlan E G Rantam F A Sunarjo S Effects of bone marrow mesenchymal stem cell transplantation on tumor necrosis factor-alpha receptor 1 expression, granulosa cell apoptosis, and folliculogenesis repair in endometriosis mouse models Vet World 2021 14 07 1788 1796 34475699 10.14202/vetworld.2021.1788-1796 PMC8404130

[16] Hendarto H Widjiati, Johari S. (2014). Curcumin Supplementation to Improve Oocyte Maturation and in Vitro Fertilization Results in Endometriosis Model Mice Maj Obstet Gynecology 2014 22 02 53 57

[17] Hsu A L Townsend P M Oehninger S Castora F J Endometriosis may be associated with mitochondrial dysfunction in cumulus cells from subjects undergoing in vitro fertilization-intracytoplasmic sperm injection, as reflected by decreased adenosine triphosphate production Fertil Steril 2015 103 02 347 520 10.1016/j.fertnstert.2014.11.002 25516080

[18] Zamaraeva M V Sabirov R Z Maeno E Ando-Akatsuka Y Bessonova S V Okada Y Cells die with increased cytosolic ATP during apoptosis: a bioluminescence study with intracellular luciferase Cell Death Differ 2005 12 11 1390 1397 15905877 10.1038/sj.cdd.4401661

[19] Robker R L Richards J S Hormone-induced proliferation and differentiation of granulosa cells: a coordinated balance of the cell cycle regulators cyclin D2 and p27Kip1 Mol Endocrinol 1998 12 07 924 940 10.1210/mend.12.7.0138 9658398

[20] Alam M H Miyano T Interaction between growing oocytes and granulosa cells in vitro Reprod Med Biol 2019 19 01 13 23 10.1002/rmb2.12292 31956281 PMC6955591

[21] Sugiura K Su Y Q Diaz F J Pangas S A Sharma S Wigglesworth K Oocyte-derived BMP15 and FGFs cooperate to promote glycolysis in cumulus cells Development 2007 134 14 2593 2603 10.1242/dev.006882 17553902

[22] Imoto Y Abe Y Honsho M Okumoto K Ohnuma M Kuroiwa H Onsite GTP fuelling via DYNAMO1 drives division of mitochondria and peroxisomes Nat Commun 2018 9 01 4634 10.1038/s41467-018-07009-z 30401830 PMC6219506

[23] Vos M Lauwers E Verstreken P Synaptic mitochondria in synaptic transmission and organization of vesicle pools in health and disease Front Synaptic Neurosci 2010 2 139 10.3389/fnsyn.2010.00139 21423525 PMC3059669

[24] Dumollard R Carroll J Duchen M R Campbell K Swann K Mitochondrial function and redox state in mammalian embryos Semin Cell Dev Biol 2009 20 03 346 353 10.1016/j.semcdb.2008.12.013 19530278

[25] Hendarto H Pathomechanism of Infertility in Endometriosis. In: Endometriosis: Basic Concepts And Current Research TrendsJaneza Trdine 9, 51000 Rijeka, Croatia, Croatia,2012 343 354

[26] Sanchez A M Vanni V S Bartiromo L Papaleo E Zilberberg E Candiani M Is the oocyte quality affected by endometriosis? A review of the literature J Ovarian Res 2017 10 01 43 28701212 10.1186/s13048-017-0341-4 PMC5508680

[27] Kazuno A A Munakata K Nagai T Shimozono S Tanaka M Yoneda M Identification of mitochondrial DNA polymorphisms that alter mitochondrial matrix pH and intracellular calcium dynamics PLoS Genet 2006 2 08 e128 10.1371/journal.pgen.0020128 16895436 PMC1534079

[28] Murphy M P Mitochondrial thiols in antioxidant protection and redox signaling: distinct roles for glutathionylation and other thiol modifications Antioxid Redox Signal 2012 16 06 476 495 10.1089/ars.2011.4289 21954972

[29] Zorov D B Juhaszova M Sollott S J Mitochondrial reactive oxygen species (ROS) and ROS-induced ROS release Physiol Rev 2014 94 03 909 950 10.1152/physrev.00026.2013 24987008 PMC4101632

[30] Zhang D Keilty D Zhang Z F Chian R C Mitochondria in oocyte aging: current understanding Facts Views Vis ObGyn 2017 9 01 29 38 28721182 PMC5506767

[31] Maynard S Schurman S H Harboe C de Souza-Pinto N C Bohr V A Base excision repair of oxidative DNA damage and association with cancer and aging Carcinogenesis 2009 30 01 2 10 10.1093/carcin/bgn250 18978338 PMC2639036

[32] Ben-Meir A Burstein E Borrego-Alvarez A Chong J Wong E Yavorska T Coenzyme Q10 restores oocyte mitochondrial function and fertility during reproductive aging Aging Cell 2015 14 05 887 895 10.1111/acel.12368 26111777 PMC4568976

[33] Boucret L Chao de la Barca J M Morinière C Desquiret V Ferré-L'Hôtellier V Descamps P Relationship between diminished ovarian reserve and mitochondrial biogenesis in cumulus cells Hum Reprod 2015 30 07 1653 1664 10.1093/humrep/dev114 25994667

[34] May-Panloup P Boucret L Chao de la Barca J M Desquiret-Dumas V Ferré-L'Hôtellier V Morinière C Ovarian ageing: the role of mitochondria in oocytes and follicles Hum Reprod Update 2016 22 06 725 743 10.1093/humupd/dmw028 27562289

[35] Defrère S Lousse J C González-Ramos R Colette S Donnez J Van Langendonckt A Potential involvement of iron in the pathogenesis of peritoneal endometriosis Mol Hum Reprod 2008 14 07 377 385 10.1093/molehr/gan033 18508952

[36] Van Blerkom J Mitochondrial function in the human oocyte and embryo and their role in developmental competence Mitochondrion 2011 11 05 797 813 10.1016/j.mito.2010.09.012 20933103

